# Real-World Effectiveness and Safety of Anifrolumab in Egyptian Patients with Moderate-to-Severe Systemic Lupus Erythematosus: A Retrospective Multicenter Study

**DOI:** 10.3390/jcm15187241

**Published:** 2026-09-17

**Authors:** Samar Tharwat, Samah A. El Bakry, Shereen Abdelsalam Olwan, Fatma Fayed, Enas S. Zahran, Maie Helal, Mai M. Morsy, Abeer Abdelmonem Shahba, Rehab Elnemr, Nehad Mohamed Elshatby, Nevine Mohannad, Gehad Maghraby, Kamel Heshmat Gado, Manal Tayel, Yasmine Abu Halawa, Dina Shahin, Ahmed Yehia Ismaeel, Eiman Soliman, Eman F. Mohamed

**Affiliations:** 1 Rheumatology & Immunology Unit, Department of Internal Medicine, Faculty of Medicine, Mansoura University, Mansoura 35516, Egypt; dinashahin68@mans.edu.eg; 2 Department of Internal Medicine, Faculty of Medicine, Horus University, New Damietta 34518, Egypt; 3 Rheumatology Unit, Department of Internal Medicine, Faculty of Medicine, Ain Shams University, Cairo 11566, Egypt; samahmn72@med.asu.edu.eg; 4 Rheumatology, Rehabilitation and Physical Medicine Department, Faculty of Medicine, Tanta University, Tanta 31527, Egypt; shereenabdelsalam80@yahoo.com; 5 Rheumatology and Immunology Unit, Internal Medicine Department, Students’ Hospital, Alexandria University, Alexandria 21526, Egypt; f_omar16@alexmed.edu.eg; 6 Rheumatology and Immunology Unit, Internal Medicine Department, Faculty of Medicine, Menoufia University, Shebeen El-Kom 32511, Egypt; inas.subhi@med.menofia.edu.eg; 7 Rheumatology, Rehabilitation and Physical Medicine Department, Faculty of Medicine, Alexandria University, Alexandria 21521, Egypt; maie.helal@gmail.com (M.H.); rehab.elnemr1@alexmed.edu.eg (R.E.); n_elshatby07@alexmed.edu.eg (N.M.E.); 8 Rheumatology and Clinical Immunology Unit, Department of Internal Medicine, Faculty of Medicine, Alexandria University, Alexandria 21526, Egypt; m_morsy17@alexmed.edu.eg (M.M.M.); manal.taiel@alexmed.edu.eg (M.T.); yasmine.saad1281@alexmed.edu.eg (Y.A.H.); solimaneiman@yahoo.com (E.S.); 9 Rheumatology & Clinical Immunology Unit, Department of Internal Medicine, Faculty of Medicine, Tanta University, Tanta 31527, Egypt; abeer.shahba@med.tanta.edu.eg; 10 Rheumatology and Clinical Immunology Unit, Department of Internal Medicine, Faculty of Medicine, Alexandria University Hospitals, Alexandria University, Alexandria 21526, Egypt; nmohannad@yahoo.com; 11 Rheumatology and Clinical Immunology Unit, Internal Medicine Department, Faculty of Medicine, Cairo University, Cairo 11562, Egypt; gehad.maghraby@kasralainy.edu.eg (G.M.); gado_k@kasralainy.edu.eg (K.H.G.); 12 Internal Medicine Department, Faculty of Medicine, Beni-Suef University, Beni-Suef 62511, Egypt; ahmed_yehia@med.bsu.edu.eg; 13 Rheumatology Unit, Internal Medicine Department, Faculty of Medicine (Girls), Al-Azhar University, Cairo 11651, Egypt; emanfekry.8@azhar.edu.eg

**Keywords:** systemic lupus erythematosus, anifrolumab, Egyptian patients, type I interferon inhibitor

## Abstract

**Background and Objectives**: Real-world data on anifrolumab in Egyptian patients with moderate-to-severe systemic lupus erythematosus (SLE) remain limited. This study aimed to evaluate the real-world effectiveness and safety of anifrolumab in Egyptian patients with moderate-to-severe SLE. **Materials and Methods**: This retrospective, multicenter, observational cohort study reviewed the medical records of 80 adults with moderate-to-severe SLE who had received at least 6 doses (≥20 weeks) of intravenous anifrolumab 300 mg every 4 weeks as part of routine clinical care at 13 Egyptian tertiary rheumatology centers; medical record review and data extraction were performed between May and July 2026. Outcomes were assessed at baseline and final follow-up and included disease activity, lupus low disease activity state (LLDAS), glucocorticoid use, flare status, laboratory parameters, and adverse events. **Results**: Patients were predominantly female (98.8%), with a mean age of 30.4 ± 9.8 years. Disease activity improved, with SLEDAI-2K decreasing from 11 to 4 (*p* < 0.001), PGA from 2.7 to 0.5 (*p* < 0.001), LLDAS increasing from 0% to 51.3% (*p* < 0.001), and flares decreasing from 95.0% to 18.8% (*p* < 0.001). Glucocorticoid burden also declined, with oral glucocorticoid use falling from 95.0% to 83.8% (*p* = 0.021), median prednisone dose from 20 mg/day to 5 mg/day (*p* < 0.001), intravenous methylprednisolone use from 50.0% to 6.3% (*p* < 0.001), and 63.8% achieving prednisone doses ≤ 7.5 mg/day (*p* < 0.001). Laboratory improvement included hemoglobin, leukocyte count, lymphocyte count, platelet count, C3, C4, anti-dsDNA, C-reactive protein (CRP), and erythrocyte sedimentation rate (ESR), while serum creatinine did not change significantly (*p* = 0.904). No serious adverse events were reported. **Conclusions**: Anifrolumab was associated with significant short-term improvement in clinical and laboratory disease activity, marked steroid sparing, and reduced flare burden in Egyptian patients with moderate-to-severe SLE, with acceptable short-term tolerability.

## 1. Introduction

Systemic lupus erythematosus (SLE) is a chronic, heterogeneous autoimmune disease characterized by relapsing-remitting inflammation and multiorgan involvement, leading to substantial morbidity and treatment burden [1]. Current understanding of SLE pathogenesis identifies type I interferon and autoreactive immune responses as central drivers of disease initiation and perpetuation [2]. Elevated type I interferon activity is associated with greater disease activity and more severe manifestations, including nephritis, which has made this pathway an attractive therapeutic target in SLE [3].

Despite advances in standard therapy, the management of moderate-to-severe SLE remains challenging. Conventional treatment with glucocorticoids and immunosuppressive agents can control inflammation, but prolonged exposure is associated with significant toxicity, including infections, organ damage, and mortality risk [4]. In addition, drug development in SLE has historically been hindered by disease heterogeneity, difficulties in trial design, and limited robustness of outcome measures [5]. These limitations have driven the development of biologic therapies that target key pathogenic pathways more selectively [6].

Anifrolumab, a fully human monoclonal antibody directed against subunit 1 of the type I interferon receptor, blocks signaling from all type I interferons and has emerged as an important targeted therapy for moderate-to-severe SLE [7]. In the phase IIb MUSE trial, anifrolumab significantly reduced disease activity across composite and organ-specific endpoints, with greater benefit observed in patients with a high interferon gene signature [8]. Subsequent phase III trials provided further support for its efficacy, particularly in BICLA response, glucocorticoid reduction, and cutaneous outcomes, while long-term extension data suggested a favorable benefit-risk profile with no major new safety signals [9].

Pooled and post hoc analyses have shown that anifrolumab improves disease activity across multiple organ domains, especially mucocutaneous, musculoskeletal, and immunological manifestations, which are among the most frequent clinical features of active SLE [10]. Early real-world studies have also suggested meaningful reductions in disease activity and corticosteroid use, with particularly notable benefit in cutaneous disease [11,12]. However, the currently available real-world evidence remains limited by relatively small sample sizes, short follow-up periods, observational designs, lack of comparator groups, and methodological heterogeneity, which constrain generalizability and causal inference [11].

These limitations are particularly important in the Middle East and North Africa (MENA), where SLE imposes a substantial clinical burden and where populations remain underrepresented in global studies of emerging biologic therapies [13]. In Egypt specifically, a nationwide study of 3661 adult patients demonstrated a broad spectrum of clinical and immunological disease expression, underscoring the need for locally relevant therapeutic data [14]. A regional review has emphasized that limited registries, unequal access to advanced therapies, and possible genetic, immunologic, and environmental differences may affect treatment response and safety, making extrapolation from international cohorts incomplete for Arab populations [13]. In addition, real-world data regarding anifrolumab from the MENA region remains sparse and has not been adequately characterized in Egyptian patients.

So, the present study was designed to evaluate the real-world effectiveness and safety of anifrolumab in Egyptian patients with moderate-to-severe SLE treated in routine clinical practice. The study specifically aimed to assess changes in clinical and laboratory disease activity, attainment of lupus low disease activity state, glucocorticoid-sparing effects, flare outcomes, and treatment tolerability in a multicenter Egyptian cohort.

## 2. Materials and Methods

### 2.1. Study Design and Settings

This multicenter, retrospective, observational cohort study was conducted at 13 tertiary rheumatology centers and units across Egypt. The study timeline consisted of two phases: (1) a variable treatment phase during which patients received anifrolumab as part of routine clinical care, with initiation dates ranging from July 2025 to May 2026; and (2) a data extraction phase performed between May and July 2026, during which investigators reviewed medical records and extracted data. Patients were eligible for inclusion if they had already completed at least 20 weeks of treatment (≥6 doses) by the time of data collection. Medical records were reviewed and data were extracted; anifrolumab treatment itself had been initiated at variable, individual dates prior to this review period, since eligibility required that each patient had already completed at least 6 doses of treatment (≥20 weeks) by the time of data collection. Patients were eligible for inclusion if they were 18 years or older at anifrolumab initiation, fulfilled the 2019 EULAR/ACR classification criteria for SLE [15], had moderate-to-severe SLE at baseline, had received at least six doses of intravenous anifrolumab 300 mg every 4 weeks as part of routine clinical care for SLE, had baseline clinical data available within the 1 month preceding the first infusion, and had at least one follow-up assessment performed at least 20 weeks after anifrolumab initiation (hereafter, the “final follow-up visit”). Moderate-to-severe SLE was defined as having a baseline SLEDAI-2K score of ≥6 and/or clinically significant organ involvement [16]. We excluded patients with severe, active proliferative nephritis (defined as proteinuria > 3.5 g/day or rapidly progressive glomerulonephritis requiring intensive induction therapy) to minimize confounding from potent concurrent immunosuppression and to focus on the drug’s effect on extra-renal and stable renal manifestations.

### 2.2. Ethical Consideration

This study was conducted in accordance with the ethical principles of the Declaration of Helsinki. The study protocol received central approval from the Institutional Review Board of the Faculty of Medicine, Mansoura University, Egypt (Approval No. R.26.05.3699), which covered the multicenter study procedures. Written informed consent for study participation and use of retrospective medical-record data was obtained from all participants prior to inclusion during their regular follow-up visits.

### 2.3. Sample Size Calculation

**Figure 1:**
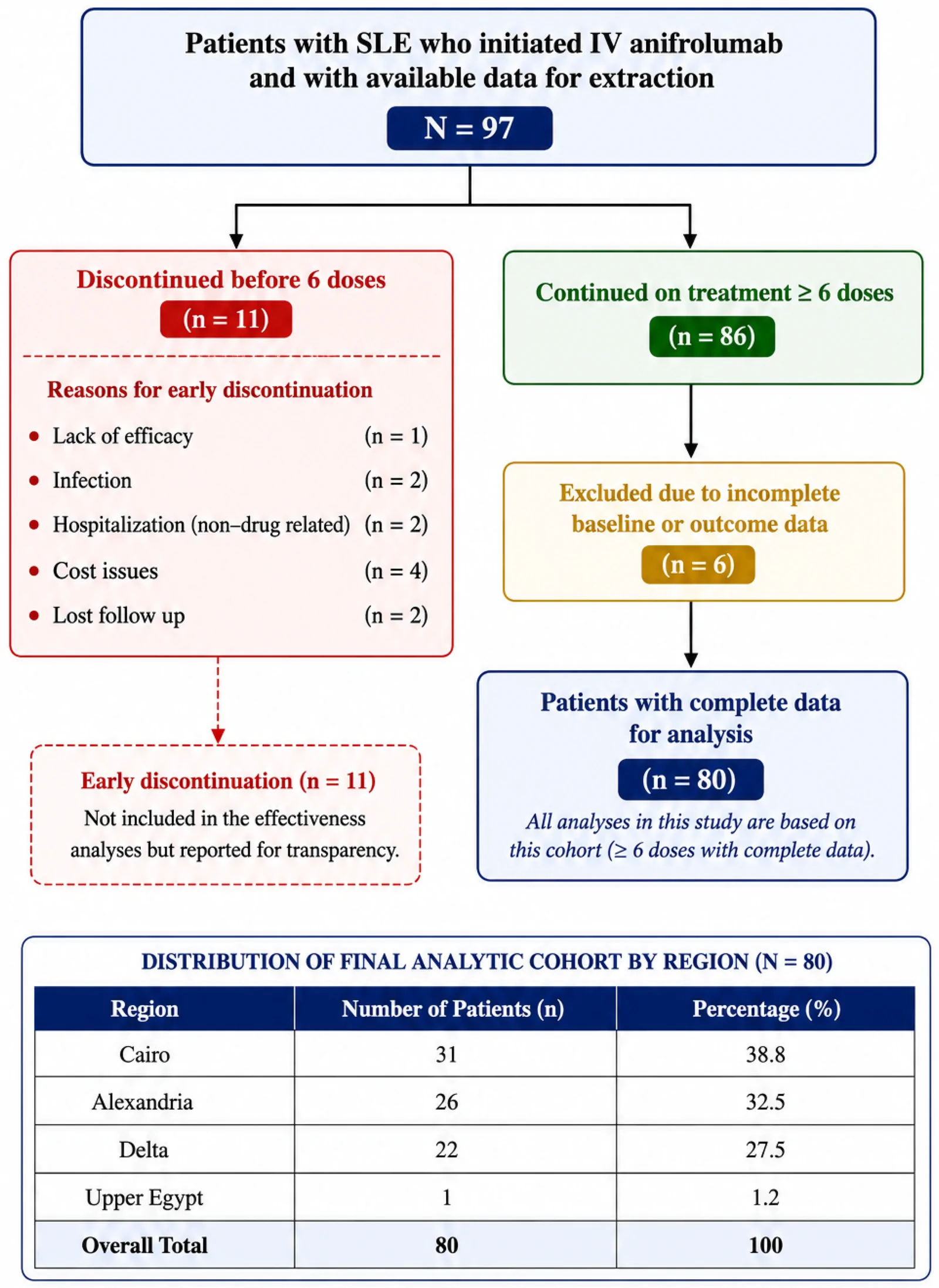
Flowchart of the study.

No formal priori sample size calculation was performed because this was a retrospective study. Patient inclusion was feasibility-based and depended on the availability of eligible Egyptian patients with SLE who received anifrolumab in routine clinical practice, as well as the completeness of their medical records for analysis. During the study period, 97 adult patients with SLE initiated intravenous anifrolumab. Of these, 11 discontinued anifrolumab before completing the prespecified 20 weeks treatment period, while 6 had incomplete baseline or outcome data. These 17 patients were not included in the primary 6-month effectiveness analysis. However, all 97 treatment initiators constituted the safety population, and available safety data were retained irrespective of treatment duration. The primary treatment-persistent effectiveness cohort therefore comprised 80 patients who completed at least 20 weeks of treatment and had complete effectiveness data (Figure 1).

### 2.4. Baseline Sociodemographic Data and Clinical Characteristics

Baseline sociodemographic and clinical characteristics were extracted from patients’ records at the time of anifrolumab initiation. This included age, sex, age at SLE diagnosis, disease duration, cumulative clinical manifestations, and cumulative organ system involvement, including cutaneous, musculoskeletal, neuropsychiatric, serosal, hematologic, and renal disease, together with specific mucocutaneous features and cutaneous lupus subtypes. Cumulative disease manifestations recorded at entry represented all organ involvement experienced since initial SLE diagnosis. Active baseline disease was clinically defined as organ-specific activity present at the time of anifrolumab initiation, which directly formed the indications for treatment initiation. Refractory disease that necessited anifrulimab was defined as persistent organ-specific clinical activity (SLEDAI-2K domain score ≥ 2) despite standard-of-care therapy, which included optimized hydroxychloroquine and at least one conventional immunosuppressive agent (e.g., mycophenolate mofetil or methotrexate) administered for at recommended doses. Steroid dependence was defined as the inability to reduce daily oral prednisone due to disease reactivation.

Renal involvement at baseline represented stable low-grade proteinuria (below 3.5 g/day or chronic renal sequelae without severe active proliferative lupus nephritis (as active proliferative nephritis requiring induction therapy was an exclusion criterion). CNS manifestations represented chronic SLE-attributed refractory headaches and mild cognitive dysfunction without acute severe neuropsychiatric lupus events (e.g., psychosis or active seizures).

Baseline therapies were defined as any immunosuppressive or biologic agent used within the 4 weeks preceding anifrolumab initiation, as documented in the medical records. For patients with prior rituximab, cyclophosphamide, or intravenous methylprednisolone exposure, the most recent documented dose was considered, provided that a minimum washout period of 6 months for rituximab and 3 months for cyclophosphamide and intravenous methylprednisolone had elapsed before anifrolumab initiation.

Additional anifrolumab treatment characteristics included whether anifrolumab was used as the first biologic therapy, the reasons for treatment initiation, and the interval between SLE onset and anifrolumab initiation.

### 2.5. Two-Time-Point Design

A two-time-point study design was used, with data collected at baseline and at the final follow-up visit. Baseline was defined as the clinical and laboratory assessment performed closest to the first (index) anifrolumab infusion, provided that it occurred within the preceding 1 month. If more than one assessment was available during this period, the assessment closest to the index infusion was selected. The final follow-up visit was defined a priori as the clinical assessment closest to 20 weeks after the first (index) infusion, provided that the assessment occurred within the prespecified 20–32-week window after treatment initiation. If more than one assessment was available within this window, the assessment with the smallest absolute deviation from week 20 was selected as the final follow-up visit. At both time points, laboratory parameters and disease activity measures were recorded, including hematologic indices, complement levels, anti-dsDNA antibodies, serum creatinine, inflammatory markers, systemic lupus erythematosus disease activity index 2000 (SLEDAI-2K) [17], physician global assessment (PGA) [18], lupus low disease activity state (LLDAS), flare status, and steroid use outcomes. PGA was scored on the standard 0–3 visual analog scale (0 = no disease activity, 1 = mild, 2 = moderate, 3 = severe) [18]. LLDAS was defined according to the validated Asia-Pacific Lupus Collaboration criteria [19], requiring all of: SLEDAI-2K ≤ 4 with no activity in major organ systems and no haemolytic anemia or gastrointestinal activity; no new features of disease activity versus the previous assessment; PGA ≤ 1; prednisolone (or equivalent) dose ≤ 7.5 mg/day; and well-tolerated standard maintenance immunosuppressive or biologic therapy. LLDAS was assessed cross-sectionally at baseline and at the final follow-up visit, reflecting the patient’s status at each single visit rather than a sustained state across the intervening period. Adverse events and infections were recorded throughout follow-up. Complement (C3, C4) and anti-dsDNA antibody levels were measured locally at each of the participating centers using the assay platform routinely used in that center’s clinical laboratory. Because assay methodology and reference thresholds are not directly interchangeable across centers, complement and anti-dsDNA results were analyzed and reported as categorical status (low C3/C4, positive anti-dsDNA) using each center’s own local, assay-specific reference range.

DORIS remission was additionally assessed at the final follow-up according to the internationally proposed DORIS remission framework [20]. Patients fulfilling the DORIS remission definition at the final assessment were classified as having achieved remission.

A flare was ascertained retrospectively from documentation in the medical record and defined as a physician-documented increase in disease activity necessitating therapeutic escalation (e.g., glucocorticoid dose increase, initiation or switch of immunosuppressive therapy, or hospitalization), assessed relative to the patient’s status at the immediately preceding clinical assessment. Baseline flares were defined as ≥1 flare in the 6 months prior to anifrolumab initiation (n=80). Follow-up flares represented the cumulative proportion of patients with ≥1 flare during the entire treatment period (n=80).

All disease-activity measures were abstracted from the medical record by a single trained investigator at each center/the treating rheumatologist, using a standardized data-extraction form; the “no new activity” component of LLDAS was determined by comparing the final-visit assessment against the immediately preceding documented assessment.

Disease activity scores (SLEDAI-2K and PGA) were recorded contemporaneously in medical records during routine clinical visits. Data abstraction was performed retrospectively by the treating rheumatologist at each participating center using a standardized data extraction form. Patients with incomplete baseline or missing component data required to calculate SLEDAI-2K, LLDAS, or DORIS criteria were excluded from the effectiveness analysis (*n* = 6). To maintain multi-center consistency, a virtual consensus meeting was held among all site investigators prior to data collection to standardize data extraction rules.

All clinical and laboratory data were extracted directly from electronic or paper health records by a designated co-author/treating rheumatologist at each center using a uniform data extraction sheet, with centralized data auditing and cross-checking performed by the principal investigator to ensure data consistency across sites.

The ‘no new features of disease activity’ component of LLDAS was determined by comparing the final follow-up clinical assessment against the patient’s documented clinical status at the immediately preceding routine visit (median interval: 4 weeks).

Classification of LLDAS and DORIS remission was based on individual patient-level data, directly derived from the documented clinical domain scores, exact steroid doses, and concurrent laboratory profiles for each patient.

### 2.6. Safety Assessment

Adverse events were ascertained retrospectively from the medical record by the treating rheumatologist at each center and coded by system organ class and preferred term. Seriousness was classified using ICH E2A/CIOMS criteria [21], under which death, a life-threatening course, hospitalization, persistent or significant disability, congenital anomaly, or another medically important outcome each independently qualify an event as serious; hospitalization was therefore a sufficient but not the only criterion, so a non-hospitalized event could still be classified as serious if it met one of the other criteria. Severity (mild, moderate, severe) was graded by the treating physician based on the clinical impact recorded in the chart. Relatedness to anifrolumab (unrelated, unlikely, possible, probable, or certain) was assessed by the treating physician using the WHO-UMC causality categories [22], informed by Naranjo-algorithm considerations [23], including the temporal relationship to infusion, the plausibility of alternative explanations, and dechallenge/rechallenge information where available. All follow-up was conducted exclusively at the participating centers.

### 2.7. Statistical Analysis

We analyzed the acquired data using SPSS version 26 (IBM Corp., Armonk, NY, USA). Normality of continuous variables was formally assessed using the Shapiro–Wilk test. Continuous variables were expressed as mean ± standard deviation for parametric data and as median (min–max) for nonparametric data, whereas categorical variables were presented as frequencies and percentages. Paired comparisons were used: the paired *t*-test for normally distributed continuous variables, the Wilcoxon signed-rank test for non-normally distributed continuous variables, and McNemar’s test for paired categorical variables. The primary effectiveness endpoint was prespecified as the change in SLEDAI-2K score from baseline to the final follow-up visit; all other outcomes (PGA, LLDAS/DORIS remission, glucocorticoid-sparing measures, laboratory parameters, and flare rate) were treated as secondary or exploratory. The patient-level change in SLEDAI-2K was calculated and reported as the median within-patient change with its 95% confidence interval. Response proportions were reported with 95% confidence intervals. Exploratory logistic regression analyses were performed to examine associations between baseline characteristics and LLDAS achievement. Given the limited number of non-LLDAS events, multivariable modeling was restricted to a small number of clinically prespecified variables, and the results were interpreted as exploratory associations rather than definitive independent predictors. Odds ratios with 95% confidence intervals were reported, and statistical significance was set at *p* < 0.05.

## 3. Results

During the study period, 97 patients initiated anifrolumab. Eleven patients discontinued treatment before completing 20 weeks, and six additional patients had incomplete effectiveness data. Accordingly, 80 patients constituted the treatment-persistent cohort for the primary effectiveness analysis.

Table 1 shows that the study included 80 patients with SLE with a mean age of 30.4 ± 9.8 years and a strong female predominance (79/80, 98.8%), while the mean age at diagnosis was 24.2 ± 9.3 years and median disease duration was 5 years (range 1–20). The most frequent cumulative clinical manifestations were musculoskeletal involvement in 76.3%, cutaneous involvement in 71.3%, oral ulcers in 62.5%, alopecia in 55.0%, and hematologic involvement in 52.5% of patients.

Table 2 shows that anifrolumab was used as the first biologic in 73.8% of patients, with treatment initiated at a median of 33 months after SLE onset. The main reasons for starting anifrolumab were refractory musculoskeletal manifestations (48.8%), cutaneous disease (43.8%), and steroid dependence (33.8%), while hematologic refractory disease accounted for 23.8% of initiations. Less frequent indications included serositis (5.0%), CNS-refractory disease (2.5%), and intolerance to prior treatment (3.8%).

Table 3 demonstrates a marked steroid-sparing effect, with oral glucocorticoid use falling from 95.0% to 83.8%, median prednisone dose decreasing from 20 mg/day to 5 mg/day, and intravenous methylprednisolone use dropping from 50.0% to 6.3%. Concomitant immunosuppressive use also declined, including cyclophosphamide, rituximab, methotrexate, and mycophenolate, while hydroxychloroquine and tacrolimus remained largely unchanged. Laboratory parameters improved in parallel, with higher hemoglobin, leukocyte, lymphocyte, and platelet counts and lower frequencies of low C3, low C4, and elevated anti-dsDNA, alongside reductions in C-reactive protein (CRP) and erythrocyte sedimentation rate (ESR). The median SLEDAI-2K score decreased from 11 at baseline to 4 at final follow-up, with a median within-patient change of −8 points (95% CI, −12.3 to −9.0; *p* < 0.001). LLDAS was achieved by 41/80 patients (51.3%; 95% CI, 39.8–62.6) at final follow-up, while DORIS remission was achieved by 14/80 patients (17.5%; 95% CI, 9.9–27.5) at final follow-up.

During treatment, 15/80 patients (18.8%) experienced a flare, compared to 76/80 (95.0%) at baseline (*p* < 0.001).

Safety outcomes were assessed in all 97 anifrolumab initiators. Adverse events were reported in 17 patients (17.5%), with pneumonia occurring in 3 (3.09%) and urinary tract infection in 2 (2.06%); other individual adverse events occurred in 1 patient each (1.03%). Eleven patients discontinued treatment before 24 weeks because of infection (3.09%), lack of efficacy (2.06%), hospitalization (2.06%), cost/access barriers (2.06%), or loss to follow-up (2.06%). No serious adverse events or deaths were recorded. Detailed safety outcomes and reasons for early discontinuation are presented in Table 4.

Table 5 shows the univariate and multivariable logistic regression analyses of predictors of LLDAS achievement. On univariate analysis, age, daily prednisone dose, mycophenolate use, anti-dsDNA positivity, SLEDAI-2K, and musculoskeletal-refractory disease were significantly associated with LLDAS (*p* < 0.05). On multivariable analysis, only daily prednisone dose (OR 0.952, *p* = 0.024) and musculoskeletal-refractory disease as the indication for anifrolumab (OR 3.185, *p* = 0.039) remained independent predictors.

## 4. Discussion

In this real-world multicenter Egyptian cohort, anifrolumab was associated with substantial clinical improvement in patients with moderate-to-severe SLE, with parallel reductions in global disease activity, flare frequency, and glucocorticoid exposure. These findings are consistent with the biologic rationale for interferon-pathway blockade in SLE, where type I interferon is a central driver of persistent inflammation and more severe disease expression.

The sharp rise in LLDAS attainment observed in our cohort, from 0% at baseline to 51.3% at follow-up, represents one of the most clinically meaningful findings of this study, given that LLDAS is now widely accepted as a realistic, validated treat-to-target endpoint in SLE that was developed after the original remission criteria proved too restrictive for routine practice [19]. This level of attainment compares favorably with outcomes reported in other real-world anifrolumab cohorts suggesting potential added value of interferon-pathway blockade in achieving stringent disease control targets [11,13]. The importance of this outcome extends beyond a numerical endpoint, since sustained LLDAS has been consistently linked to protection against organ damage accrual, reduced flare risk, and better health-related quality of life across multiple international cohorts [19,24]. However, differences in patient selection, concomitant treatment, endpoint ascertainment, and follow-up duration preclude any comparative conclusion that anifrolumab exceeds conventional therapy or confers added therapeutic value relative to other treatments, and no such comparison was formally tested in our study.

Our findings are broadly consistent with the pivotal phase III TULIP program while extending those observations into routine clinical practice. In TULIP-1, anifrolumab did not meet the primary SRI-4 endpoint at week 52, but several secondary and organ-specific outcomes favored treatment, including BICLA response, cutaneous improvement, and glucocorticoid tapering [25]. In TULIP-2, where BICLA was selected as the primary endpoint, anifrolumab showed superior overall response versus placebo at week 52, with additional benefits in glucocorticoid reduction and skin disease severity [16]. In the present study, the SLE cohort demonstrated a marked steroid-sparing effect after anifrolumab initiation, with median prednisone dose decreasing from 20 mg/day to 5 mg/day, 73.8% of patients achieving at least 50% steroid reduction, and 63.8% reaching ≤7.5 mg/day by follow-up, alongside parallel declines in cyclophosphamide, rituximab, methotrexate, and mycophenolate. This finding is consistent with phase III trial data, where anifrolumab improved sustained glucocorticoid tapering compared with placebo and allowed dose reduction to ≤7.5 mg/day without loss of disease control [16,26]. Comparable steroid-sparing benefits have also been reported in real-world cohorts from Spain [11], Denmark [27], Japan, Toronto [28], and Italy [29], where prednisone or prednisolone doses declined rapidly, often by more than 50%, while disease activity simultaneously improved. 

In the Japanese LOOPS registry, anifrolumab was associated with lower glucocorticoid exposure than standard care in patients with minor flares, suggesting that interferon blockade may reduce reliance on steroid escalation in routine practice [30]. Smaller real-world series further support this pattern, with successful corticosteroid tapering observed in all treated patients and, in some cases, complete discontinuation within months [31]. Collectively, these findings reinforce that anifrolumab can facilitate meaningful reduction in both glucocorticoids and concomitant immunosuppressive burden, which is clinically important given current treatment goals that prioritize disease control while minimizing long-term steroid toxicity [32].

In the present cohort, anifrolumab was associated with marked improvement in laboratory and clinical activity measures, including higher hemoglobin, leukocyte, lymphocyte, and platelet counts; lower frequencies of low C3, low C4, and elevated anti-dsDNA; reduced CRP and ESR; and major declines in SLEDAI-2K and PGA, alongside a rise in LLDAS from 0% to 51.3% and a fall in flares from 95.0% to 18.8%. Similar patterns were reported in the largest real-world Spanish cohort, where disease activity scores improved from the first month and serological markers showed progressive C3/C4 recovery with declining anti-dsDNA titers [11]. The absence of significant change in serum creatinine and the stability of low-grade proteinuria in this cohort are consistent with the study’s baseline exclusion of patients with severe, active proliferative nephritis.

Our findings also align with other real-world studies showing rapid declines in SLEDAI-2K and sustained low disease activity or remission, including Italian, Danish, German, Japanese, and Toronto cohorts [27,29]. Smaller series further support improvement in hematologic and inflammatory parameters, with normalization of lymphopenia, improvement of cytopenias, and reduction in ESR, although serologic responses were not fully consistent across all cohorts [33]. This overall pattern is also concordant with phase III trial evidence showing broader disease-activity benefit with anifrolumab and with comparative real-world data suggesting reductions in SLEDAI-2K, glucocorticoid use, and anti-DNA titers that are broadly similar to belimumab [16,34]. This comparison, however, is descriptive rather than direct, since the belimumab and anifrolumab cohorts referenced differ in baseline patient characteristics, outcome definitions, concomitant immunosuppressive use, and length of follow-up; no formal statistical or matched comparison between the two agents was undertaken in the present study, and these observations should not be interpreted as evidence of comparative efficacy. Overall, our study supports the idea that anifrolumab was associated with meaningful improvements in laboratory parameters and disease activity in routine care, with the wider literature showing reproducible benefit across multiple cohorts, although the magnitude of serologic improvement appears to vary by sample size and follow-up duration.

Overall, the observed safety findings in our cohort were limited in frequency, although these findings should be interpreted cautiously given the retrospective design and potential for under-ascertainment. These findings are broadly consistent with the larger Spanish real-world cohort, in which adverse events occurred in 13.1% of patients and serious infections in 3.9%, with herpes zoster, respiratory infections, and headache being the most frequent events [11]. Similar real-world studies from Italy, Denmark, and Toronto also described an overall acceptable or good tolerability profile, although herpesvirus-related infections remained the most recurrent safety signal and occasional severe cases were reported. The Egyptian results also align with phase III trial data showing that most adverse events were mild to moderate and that infusion-related or hypersensitivity reactions were uncommon, while herpes zoster occurred more often with anifrolumab than with placebo [16]. TULIP-1 similarly found that herpes zoster was increased but usually cutaneous and self-limited, and that infusions were generally well tolerated [26]. Overall, the available evidence supports that anifrolumab is generally well tolerated in routine care and trials, but careful monitoring for infections, particularly viral infections such as herpes zoster, remains warranted [11,34,35].

A key strength of the present study is its multicenter real-world design, which captured routine use of anifrolumab across tertiary rheumatology centers in Egypt. Another strength is the comprehensive assessment of clinically relevant outcomes, including disease activity, LLDAS attainment, flare burden, glucocorticoid sparing, laboratory response, and safety, which provides a broad view of treatment effectiveness in daily practice. However, the study is limited by its retrospective observational design, which restricts causal inference and increases susceptibility to selection and information bias. The uncontrolled before-after design precludes causal attribution of the observed improvements to anifrolumab specifically. Additional limitations include the relatively small sample size, feasibility-based recruitment without a formal priori sample size calculation, and exclusion of some patients because of incomplete records or short treatment duration. Requiring completion of at least 6 anifrolumab doses for inclusion may have introduced survivorship/selection bias, as patients who discontinued treatment earlier because of adverse events or inefficacy were excluded; this likely favors more optimistic effectiveness and safety estimates than would be seen in an all-comers population. The minimum follow-up duration of 20 weeks and the lack of a comparator group also limit assessment of long-term durability, rare adverse events, and comparative effectiveness versus other biologic or standard therapies. Several immunosuppressive agents (cyclophosphamide, methotrexate, mycophenolate) were also reduced during follow-up alongside glucocorticoid tapering. As these changes occurred concurrently with anifrolumab initiation in this non-randomized design, they are a potential confounder, and the observed improvement cannot be attributed to anifrolumab alone. An additional limitation is the absence of validated, organ-specific outcome measures. Flare ascertainment relied on unblinded, non-centralized physician documentation in the medical record rather than a validated flare instrument, which may introduce ascertainment variability between centers and physicians. Finally, the absence of serious adverse events should be interpreted cautiously given the modest cohort size and short follow-up, which limit power to detect rare or delayed safety signals; larger, longer-term studies are needed to confirm the safety profile.

Nevertheless, the study provides important locally relevant evidence on anifrolumab use in Egyptian patients with moderate-to-severe SLE and offers a useful foundation for larger prospective controlled studies in the region.

## 5. Conclusions

In conclusion, anifrolumab was associated with meaningful clinical improvement in Egyptian patients with moderate-to-severe SLE, with marked reductions in disease activity, flare burden, and glucocorticoid exposure during routine practice. The high rate of LLDAS attainment (51.3%), the achievement of DORIS remission in 17.5% and the strong steroid-sparing effect suggest that anifrolumab can help achieve clinically relevant treat-to-target goals beyond controlled trial settings, among patients who persisted on treatment; these findings should be interpreted with the survivorship bias inherent to retrospective, dose/duration-conditioned cohort designs in mind. These findings are clinically important because reducing long-term steroid dependence remains a central objective in SLE management, and anifrolumab treatment was associated with reduction in concomitant immunosuppressive burden. The observed safety findings were reassuring within this selected cohort, although they should be interpreted cautiously because patients with early discontinuation were not included in the final analytic cohort. Taken together, these results help define the place of anifrolumab in Egypt.

## Figures and Tables

**Table 1:** Baseline Demographic and Clinical Characteristics of Patients Included and Not Included in the Primary Effectiveness Analysis.

Variable Mean ± SD, *n* (%), Median (Min–Max)	Included in Primary Effectiveness Analysis (*n* = 80)	Not Included (*n* = 17)
Age, years	30.4 ± 9.8	32.2 ± 10.00
Age at SLE Diagnosis	24.2 ± 9.3	24.4 ± 5.9
Sex		
Female	79 (98.8)	17 (100)
Male	1 (1.3)	0
Disease duration, years	5 (1–20)	5 (1–23)
Cumulative clinical manifestations (Ever occurred)		
Cutaneous Involvement		
ACLE	32 (40)	4 (23.5)
SCLE	6 (7.5)	2 (11.8)
CCLE/DLE	24 (30)	6 (35.3)
Alopecia	44 (55)	6 (35.3)
Oral Ulcers	50 (62.5)	5 (29.4)
Other	5 (6.3)	3 (17.6)
Musculoskeletal Involvement		
Arthritis	49 (61.3)	10 (58.8)
Arthralgia	17 (21.3)	4 (23.5)
Neuropsychiatric	9 (11.3)	1 (5.9)
Serositis	23 (28.8)	2 (11.8)
Hematologic Involvement	42 (52.5)	7 (41.2)
Renal Involvement		
Nephrotic Syndrome	10 (12.5)	5 (29.4)
Nephritic Syndrome	10 (12.5)	4 (23.5)

SLE, systemic lupus erythematosus; ACLE, acute cutaneous lupus erythematosus; SCLE, subacute cutaneous lupus erythematosus; CCLE, chronic cutaneous lupus erythematosus; DLE, discoid lupus erythematosus.

**Table 2:** Anifrolumab treatment details and indications in the study SLE patients (*n* = 80).

Variable Mean ± SD, *n* (%), Median (Min–Max)	Total Cohort (*n* = 80)
Anifrolumab as first biologic	59 (73.8)
Reason for anifrolumab Initiation *	
Cutaneous refractory	35 (43.8)
Musculoskeletal refractory	39 (48.8)
Hematologic refractory	19 (23.8)
Steroid dependence	27 (33.8)
Serositis	4 (5.0)
Associated CNS refractory	2 (2.5)
Intolerance to prior treatment	3 (3.8)
Months from SLE onset to anifrolumab	33 (3–180)

SLE, systemic lupus erythematosus; CNS, central nervous system. * Note: Reasons for anifrolumab initiation reflect the active baseline refractory organ manifestations and clinical indications present at treatment start. The median time from anifrolumab initiation to the final follow-up visit was 20 (range 20–32 weeks); patients had received a median of 6 infusions (range 6–9) by the final visit.

**Table 3:** Immunosuppressive/biologic therapies, laboratory data and disease activity and response at the baseline and final visit in the study SLE patients (*n* = 80).

Variable Mean ± SD, *n* (%), Median (Min–Max)	Baseline (*n* = 80)	Final Visit * (*n* = 80)	*p*
Concomitant treatment			
Oral Glucocorticoid	76 (95.0)	67 (83.8)	0.021
Daily prednisone dose, mg/day	20 (0–60)	5 (0–30)	<0.001
Methotrexate	31 (38.8)	15 (18.8)	<0.001
Mycophenolate	63 (78.8)	49 (61.3)	<0.001
Azathioprine	13 (16.3)	9 (11.3)	0.581
Hydroxychloroquine	75 (93.8)	72 (90.0)	0.508
Tacrolimus	8 (10.0)	5 (6.3)	0.250
Treatment at pre-baseline			
IV Methylprednisolone (3 months earlier)	40 (50.0)	5 (6.3)	<0.001
Cyclophosphamide (3 months earlier)	7 (8.8)	1 (1.3)	0.031
Rituximab (6 months earlier)	12 (15.0)	0	0.001
Laboratory data			
Hemoglobin (g/dL)	10.2 ± 1.7	11.5 ± 1.2	<0.001
Leukocyte Count (×10^9^/L)	4.7 ± 1.8	6.0 ± 2.0	<0.001
Lymphocyte Count (×10^9^/L)	1.6 (0.30–4.0)	2.1 (0.6–4.9)	<0.001
Platelet Count (×10^9^/L)	224.1 ± 95.7	253.6 ± 84.7	0.005
Low C3	55 (68.8)	7 (8.8)	<0.001
Low C4	50 (62.5)	9 (11.3)	<0.001
High Anti-dsDNA	60 (75.0)	18 (22.5)	<0.001
Serum Creatinine (mg/dL)	0.9 ± 0.3	0.9 ± 0.2	0.904
Proteinuria	22 (27.5)	19 (23.8)	0.648
CRP (mg/L)	12.0 (2.1–96.0)	5 (1–31)	<0.001
ESR (mm/hr)	60 (12–125)	24 (5–96)	<0.001
Disease Activity and Response			
SLEDAI-2K	11 (7–46)	4 (0–26)	<0.001
PGA	2.7 (1–3)	0.5 (0–3)	<0.001
LLDAS	0	41 (51.3)	<0.001
DORIS remission	0	14 (17.5)	0.001
Flares ^&^	76 (95.0)	15 (18.8)	<0.001
Steroid reduction ≥ 50%	0	59 (73.8)	<0.001
Patients achieving ≤ 7.5 mg/day	22 (27.5)	51 (63.8)	<0.001

* Final Visit: assessment performed at the final follow-up visit, i.e., ≥20 weeks after anifrolumab initiation. ^&^ flare during the 24 weeks preceding anifrolumab initiation. IV, intravenous; C3, complement component 3; C4, complement component 4; anti-dsDNA, anti-double-stranded DNA antibodies; CRP, C-reactive protein; DORIS, Definition of Remission in SLE; ESR, erythrocyte sedimentation rate; SLEDAI-2K, Systemic Lupus Erythematosus Disease Activity Index 2000; PGA, Physician Global Assessment; LLDAS, Lupus Low Disease Activity State.

**Table 4:** Safety, Adverse Events, and Treatment Discontinuation Among All Anifrolumab Treatment Initiators (*n* = 97).

Adverse Event *n* (%)	Patients with Event (*n* = 97)
Any Adverse Event	17 (17.5)
Gastrointestinal disorders	
Nausea	1 (1.03)
General disorders and administration site conditions	
Body aches	1 (1.03)
Infusion reaction	1 (1.03)
Thirst	1 (1.03)
Fatigue	1 (1.03)
Immune system disorders	
Angioedema	1 (1.03)
Infections and infestations	
Herpes zoster	1 (1.03)
Pneumonia	3 (3.09)
Urinary tract infection	2 (2.06)
Other infections	1 (1.03)
Musculoskeletal and connective tissue disorders	
Worsening arthritis	1 (1.03)
Nervous system disorders	
Severe headache	1 (1.03)
Eye disorders	
Blurring of vision	1 (1.03)
Skin and subcutaneous tissue disorders	
Rash	1 (1.03)
Reasons for Early Treatment Discontinuation	
Infection	3 (3.09)
Lack of efficacy	2 (2.06)
Hospitalization	2 (2.06)
Cost/access barriers	2 (2.06)
Loss to follow-up	2 (2.06)
Serious Adverse Events (SAEs)	0
Deaths	0

SAEs, serious adverse events.

**Table 5:** Univariate and multivariable logistic regression analyses identifying predictors of achievement of lupus low disease activity state (LLDAS).

Variable	Univariate Analysis			Multivariable Analysis		
	OR	95% CI	*p*	OR	95% CI	*p*
Demographics and disease characteristics						
Age, years	1.061	1.010–1.116	0.019	1.033	0.977–1.093	0.256
Disease duration, years	0.110	0.807–1.022	0.908			
Baseline therapies						
Oral Glucocorticoid	1.054	0.141–7.874	0.959			
Daily prednisone dose, mg/day	0.950	0.916–0.985	0.006	0.952	0.912–0.993	0.024
Rituximab	0.259	0.064–1.048	0.058			
Methotrexate	1.445	0.579–3.605	0.430			
Mycophenolate	0.284	0.083–0.978	0.046	0.835	0.187–3.724	0.813
Azathioprine	0.340	0.040–2.854	0.320			
Hydroxychloroquine	3.429	0.341–34.478	0.295			
Tacrolimus	0.237	0.044–1.264	0.092			
Baseline laboratory parameters						
Hemoglobin, g/dL	1.216	0.932–1.587	0.150			
Leukocyte count, ×10^9^/L	0.888	0.693–1.137	0.346			
Lymphocyte count, ×10^9^/L	0.686	0.397–1.184	0.176			
Platelet count, ×10^9^/L	1.003	0.998–1.008	0.230			
Low C3	0.758	0.293–1.960	0.567			
Low C4	0.875	0.353–2.166	0.773			
High Anti-dsDNA	0.255	0.082–0.792	0.018	0.572	0.149–2.190	0.415
CRP, mg/L	1.014	0.982–1.047	0.393			
ESR, mm/hr	0.987	0.970–1.003	0.110			
Serum Creatinine (mg/dL)	0.279	0.059–1.320	0.107			
Proteinuria	0.433	0.157–1.191	0.105			
Baseline disease activity						
SLEDAI-2K	0.920	0.862–0.982	0.012	0.944	0.879–1.015	0.120
PGA	0.694	0.306–1.578	0.384			
Reason for anifrolumab initiation						
Cutaneous refractory	2.319	0.936–5.728	0.069			
Musculoskeletal refractory	2.790	1.127–6.908	0.027	3.185	1.059–9.575	0.039
Hematologic refractory	1.075	0.383–3.015	0.890			
Steroid dependence	1.298	0.512–3.292	0.583			
Serositis	0.949	0.127–7.087	0.959			
Associated CNS refractory	0.950	0.057–15.734	0.971			

LLDAS, lupus low disease activity state; SLEDAI-2K, Systemic Lupus Erythematosus Disease Activity Index 2000; PGA, Physician Global Assessment; CRP, C-reactive protein; ESR, erythrocyte sedimentation rate; Anti-dsDNA, anti-double-stranded DNA. In the multivariable logistic regression analysis, variables with *p* < 0.05 in univariate analysis were considered for inclusion in the multivariable model. Statistical significance was defined as *p* < 0.05.

## Data Availability

The datasets used and/or analyzed during the current study are available from the corresponding author on reasonable request.

## Abbreviations

The following abbreviations are used in this manuscript:

ACR	American College of Rheumatology
ANIFRO-EGYPT	Anifrolumab in Egyptian Patients with Systemic Lupus Erythematosus Study
anti-dsDNA	Anti-double-stranded DNA antibodies
BICLA	British Isles Lupus Assessment Group-based Composite Lupus Assessment
CNS	Central Nervous System
CRP	C-reactive protein
EULAR	European League Against Rheumatism
ESR	Erythrocyte sedimentation rate
LLDAS	Lupus Low Disease Activity State
MENA	Middle East and North Africa
PGA	Physician Global Assessment
SLE	Systemic Lupus Erythematosus
SLEDAI-2K	Systemic Lupus Erythematosus Disease Activity Index 2000
